# Pharmacological Approaches and Regeneration of Bone Defects with Dental Pulp Stem Cells

**DOI:** 10.1155/2021/4593322

**Published:** 2021-09-29

**Authors:** M. Adamička, A. Adamičková, L. Danišovič, A. Gažová, J. Kyselovič

**Affiliations:** ^1^Faculty of Medicine, Comenius University Bratislava, Institute of Medical Biology, Genetics and Clinical Genetics, Špitálska 24, 813 72 Bratislava, Slovakia; ^2^Faculty of Medicine, 5th Department of Internal Medicine, Comenius University Bratislava, Špitálska 24, 813 72 Bratislava, Slovakia; ^3^Faculty of Medicine, Comenius University Bratislava, Institute of Pharmacology and Clinical Pharmacology, Špitálska 24, 813 72 Bratislava, Slovakia

## Abstract

Bone defects in the craniomaxillofacial skeleton vary from small periodontal defects to extensive bone loss, which are difficult to restore and can lead to extensive damage of the surrounding structures, deformities, and limited functions. Plenty of surgical regenerative procedures have been developed to reconstruct or prevent alveolar defects, based on guided bone regeneration involving the use of autogenous bone grafts or bone substituents. However, these techniques have limitations in the restoration of morphological and functional reconstruction, thus stopping disease progression but not regenerating lost tissue. Most promising candidates for regenerative therapy of maxillofacial bone defects represent postnatal stem cells, because of their replication potential in the undifferentiated state and their ability to differentiate as well. There is an increased need for using various orofacial sources of stem cells with comparable properties to mesenchymal stem cells because they are more easily available with minimally invasive procedures. In addition to the source of MSCs, another aspect affects the regeneration outcomes. Thermal, mechanical, and chemical stimuli after surgical procedures have the ability to generate pain, usually managed with pharmacological agents, mostly nonsteroidal anti-inflammatory drugs (NSAIDs). Some studies revealed that NSAIDs have no significant cytotoxic effect on bone marrow stem cells from mice, while other studies showed regulation of osteogenic and chondrogenic marker genes in MSC cells by NSAIDs and paracetamol, but no effect was observed in connection with diclofenac use. Therefore, there is a need to focus on such pharmacotherapy, capable of affecting the characteristics and properties of implanted MSCs.

## 1. Introduction

Bone defects in the craniomaxillofacial skeleton vary from small periodontal defects to extensive bone loss resulting from injuries, surgical excision, congenital deformities, or advanced resorption of the alveolar bone after teeth loss. These defects are difficult to restore due to complex three-dimensional structural needs and can therefore lead to extensive damage of the surrounding structures with deformations and limited function [1]. The incidence of dental defects has become very common, seriously affecting the health and quality of a patient's life, along with heavy financial burden for its repair. Dental defects can be generally reconstructed with autogenous bone grafts, allograft, xenograft, isograft, or alloplastic material. Autogenous bone grafts obtained from the iliac crest, tibia, ribs, and others remain the gold standard to obtain accurate bone volume and morphology with long-term predictable results. However, all of these techniques possess certain advantages and shortcomings and have limitations in the restoration of morphological and functional reconstruction of defects [2, 3]. Moreover, current therapies can only improve clinical diagnostic parameters and stop disease progression but cannot regenerate lost tissue. Therefore, new biomedical technologies are in great demand to achieve excellent bone and dental tissue regeneration with morphological and functional restoration.

Regenerative medicine is a relatively new field of study with promising outcomes in healing tissues and organs and in restoring its function [4]. Stem cells are an autologous source of unspecialized cells with the ability to proliferate and differentiate into multiple types in the presence of internal or external signals. Mesenchymal stem cells (MSCs) obtained mostly from bone marrow after iliac crest aspiration with self-renewal and multiple differentiation potential are the most widely studied and documented source of such progenitor cells. MSCs have osteogenic, chondrogenic, adipogenic, angiogenic, and neurogenic properties, and their bone formation potential has been tested in a range of craniofacial defects [5–7]. Nonetheless, the isolation of such cells from bone marrow is an invasive procedure that limits their use.

Therefore, there is an increased need for using various orofacial sources of stem cells with oral and maxillofacial origin, which present a viable substituent to MSCs. These cells have comparable properties to MSCs and are more easily available with minimally invasive procedures [8]. But, in addition to the source of MSCs, another aspect affects the regeneration outcomes. Thermal, mechanical, and chemical stimuli after surgical procedures have the ability to generate pain, which is usually managed with pharmacological agents, mostly nonsteroidal anti-inflammatory drugs (NSAIDs). There is a need to focus on such pharmacotherapy, capable of affecting the characteristics and properties of implanted MSCs.

## 2. Bone Grafting Strategies

Reconstruction of maxillofacial bone defects is always a clinical challenge. The most common cause of alveolar bone deficiencies in the horizontal and vertical dimensions is ridge remodellation due to tooth loss [9, 10]. Another issue represents the posterior region of maxillary alveolar bone, which is complicated by pneumatization of the maxillary sinuses [11]. Furthermore, there are more challenging and complicated defects, which result from trauma, radiation/drugs-related osteonecrosis, or tumor resection. Plenty of surgical regenerative procedures have been developed to reconstruct or prevent alveolar defects, such as alveolar ridge or socket preservation following tooth extraction [12], maxillary sinus floor augmentation (maxillary sinus lift) [13], and mandibular ridge expansion using horizontal bone-splitting technique [14]. All these techniques are based on the guided bone regeneration principle and mainly involve the use of autogenous bone grafts and bone substituents—often in combination with barrier membranes [15]. In the case of larger defects, vascularized tissue flaps are required [16].

Autogenous bone (AB) transplantation is still considered the gold standard due to its osteoconductivity, osteoinductivity, and osteogenecity. In many cases, there is an insufficient offer of AB locally, leading to the need of harvesting from the second surgical site intraorally, such as mandibular ramus and the chin. The iliac crest represents the most frequently used extraoral donor site for AB harvesting. This procedure involves general anaesthesia, hospitalization, longer recovery, and significantly higher costs [17]. Moreover, AB harvesting is associated with the donor site morbidity risk, infections, postoperative pain, and prolonged recovery. Further disadvantages include unpredictable bone quality and quantity and excessive remodelling after surgery, especially without barrier membrane use. Alternatives include a variety of allogeneic, xenogeneic, and alloplastic substitutes. Their benefits include decreased operative trauma and blood loss, unlimited supply, absence of donor site morbidity, and extremely low antigenic potential [18].

Most promising candidates for regenerative therapy of maxillofacial bone defects represent postnatal stem cells, because of their replication potential in an undifferentiated state and their ability to differentiate as well [19].

## 3. Stem Cells

The stem cells used for tooth and periodontal regeneration are dental and nondental mesenchymal stem cells (MSCs). MSC, also called mesenchymal stromal cells, are adherent, fibroblast-like cells capable of self-renewal and multilineage differentiation. They were described more than a half century ago from cell cultures of murine bone marrow by Friedenstein et al., who defined them as a colony-forming unit fibroblast [20]. Today, MSCs are defined as nonhematopoietic progenitor cells with the ability to differentiate into distinct mesodermal lineages, which can produce bone, cartilage, fat or fibrous connective tissue, and others [21].

According to the Mesenchymal and Tissue Stem Cell Committee of the International Society for Cellular Therapy, minimal criteria to define multipotent mesenchymal stem cells are as follows:

- MSC must be adherent to plastic when maintained in standard culture conditions
- MSC must be positive for CD105, CD73, and CD90 markers and lack expression of CD45, CD34, CD14 or CD11b, CD79a, and human leukocyte antigen-D-related (HLA-DR) surface molecules
- MSC must differentiate into osteoblasts, adipocytes, and chondroblast *in vitro* [22]

MSCs are able to secrete a variety of biologically active molecules, including cytokines, growth factors, and chemokines, known as MSC secretome. It is believed that this MSC secretome plays a crucial role in the reparative processes, as therapeutic effects persist even if MSCs do not engraft or differentiate into tissue-specific cells [23, 24].

## 4. Role of Stem Cells in Bone Regenerative Process

Bone healing is a complex process consisting of inflammation, repair, and remodelling associated with many intracellular signalling pathways responsible for the regeneration of new bone. The exact mechanism of MSCs in bone repair remains unclear, as these cells interfere with several regenerative processes, such as homing, differentiation, apoptosis, or inflammation. MSCs can be polarized into either a proinflammatory or immunosuppressive phenotype based on toll-like receptors; moreover, they interfere with polarization of monocytes through the nuclear factor NF-*κ*B and signal transducer and activator of transcription 3 pathways. Another MSC mechanism is affecting the levels of interleukin 6, tumor necrosis factor-*α*, and interleukin 1*β*, leading to a better regeneration with inhibition of the progression of fibrosis. Moreover, the paracrine activity of MSC and their secretome contribute to the enhancement of angiogenesis, which involves a complex interaction between endothelial cells and the surrounding microenvironment, including endothelial cell survival, proliferation, migration, and tube formation [25].

## 5. Stem Cells Derived from Dental Tissues

**Figure 1:**
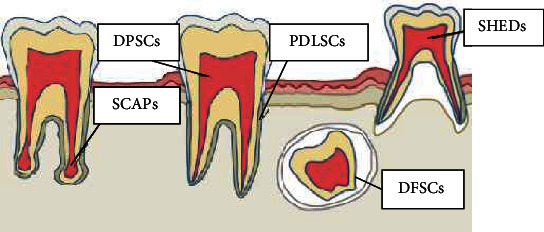
Orofacial sources of stem cells.

Stem cells isolated from teeth are more easy to obtain compared to bone marrow-derived MSCs. Sources of dental tissues with stem cells are mostly exfoliated deciduous teeth and impacted third molars, which are the most extracted teeth, together with premolars commonly extracted for orthodontic treatment [26]. Orofacial sources of stem cells are (Figure 1)

- human dental pulp stem cells (hDPSCs)
- human periodontal ligament stem cells (hPDLSCs)
- stem cells from human exfoliated deciduous teeth (SHEDs)
- stem cells from the dental apical papilla (SCAPs)
- stem cells from the dental follicle (DFSCs)

### 5.1. Human Dental Pulp Stem Cells (hDPSCs)

Isolated from the pulp with reparative and regenerative properties, hDPSCs are the first isolated MSCs from human teeth discovered by Gronthos et al. in 2000 [27]. Thanks to the ability to replenish odontoblast during restoration of dentin, hDPSCs are fundamental in postnatal tooth homeostasis and reparation. Besides, they have potential to differentiate into osteoblast, chondrocytes, myocytes, adipocyte, and neurocytes *in vitro* and *in vivo* [28]. Bone formation with use of autologous DPSCs was evaluated by d'Aquino et al. [29]. They assessed bone formation, distal to mandibular second molar, using DPSCs obtained from the extracted maxillary third molar together with collagen sponge scaffold. Present bone defect was filled with this combination on one side while the other side was considered as the control, filled with collagen scaffold. By clinical and radiographical evaluation, complete bone regeneration was observed three months postoperatively together with an increased clinical attachment levels in the experimental part compared to the control side. The patient was examined three years after surgery with qualitative compact bone in the test side, compared to the spongy bone that is physiologically found in the area [30]. Similarly, a randomized clinical study was performed by Barbier et al. in 2018 [31]. They inserted autologous DPSCs via the collagen matrix to achieve healing of postextraction socket of the mandibular third molar. They reported no differences in bone fill in the six-month follow-up. Promising results were conducted in animal models with a combination of hDPSCs with chemical agents like aspirin and aloe vera [32, 33]. A cellular model by Trubiani et al. demonstrated that stem cells from oral tissues (hPDLSCs, hDPSCs, and human gingival mesenchymal stem cells) remained the expression of surface markers related to MSC characteristics; the degree of cell proliferation rate unchanged compared to stem cells at passages 2 and 15 [34]. Their study of senescence marker expression has demonstrated the safety of transplanting long-term cultured MSCs in stem cell therapy [35].

### 5.2. Human Periodontal Ligament Stem Cells (hPDLSCs)

The periodontal ligament is a highly vascularized connective tissue located between the cementum of the root and the alveolar bone socket wall. It plays an important role in distributing the occlusal force applied to the tooth during chewing. Stem cells isolated from the periodontal ligament are named periodontal ligament stem cells and have been shown to possess similar characteristics to the bone marrow stem cells, with the potential to differentiate into adipocytes, osteoblast, and chondrocytes under specific conditions. Moreover, they have the ability to form cementum and periodontal ligament structures in surgically created periodontal defects in animal models, providing their potential for periodontal tissue regeneration [36–38]. Clinical application of autologous implantation of hPDLSCs was evaluated by Shalini et al. They implanted hPDLSCs along with its niche, and no additional bone graft material was used in healing intrabony defects. A significant reduction in probing depth together with growth in clinical attachment levels was reported compared to controls treated with open flap debridement with no graft or implantation at all [39]. Periodontal regeneration with autologous periodontal ligament-derived stem cell sheets was assessed in ten patients' study by Iwata et al. They verified the safety and efficacy of PDL-derived cell sheets isolated from extracted wisdom teeth in patients with chronic periodontitis. They transplanted three-layer PDL-derived cell sheets in an autologous fashion following standard flap surgery and filled bony defects with beta-tricalcium phosphate granules. Therapeutic effects like reduction of periodontal probing depth, clinical attachment gain, and increase of radiographic bone height were found out in all 10 cases at the 6 months after the transplantation. This approach based on cell sheet engineering proved its safety and efficacy and offers an innovative strategy for treating severe periodontal defects [40]. The immunomodulatory properties of hPDLSCs and their paracrine mechanism were revealed in a study by Diomede et al. They demonstrated that the paracrine factors secreted by hPDLSCs can accumulate in conditioned medium and thus regulate cell mobilization and osteogenic differentiation. They investigated the effect of hPDLSCs and their conditioned medium on bone regeneration with the use of a commercially available membrane scaffold Evolution (EVO) which was implanted in rat calvarias. The *in vivo* results proved that EVO membrane with hPDLSCs and conditioned medium had a better osteogenic ability to repair the calvarias defect and showed a promising therapeutic potential for application of cultured medium from hPDLSCs and scaffolds for bone defect regeneration [41].

### 5.3. Stem Cells from Human Exfoliated Deciduous Teeth (SHEDs)

SHEDs are types of human dental tissue-derived mesenchymal stem cells, possessing a capacity for self-renewal, multilineage differentiation, and immunomodulatory functions. SHEDs obtained from exfoliated deciduous teeth in mixed dentition stages of children are considered to be immature MSCs, which can be easily obtained with limited ethical and legal concerns. Compared to DPSCs, SHED exhibits a higher proliferation rate, differentiation potential, and increased mineralization *in vivo*, but studies revealed failing regeneration of a dentin-pulp complex [42]. Based on the results of previous studies, *in vitro* expansion of SHEDs is related to alterations of MSC characteristics, reduced differentiation capacity, and shortened telomeres, together with spontaneous malignant transformation [43]. Thus, the analysis of SHED characteristics in long-term cultivation needs to be studied and elucidated.

In addition to the abovementioned oral stem cells, there are other sources with promising potential properties. These include dental follicle stem cells, stem cells from apical papilla, gingiva-derived mesenchymal stem cells, and tooth germ progenitor cells, studied *in vitro* and *in vivo* in animal models.

### 5.4. Stem Cells from the Dental Apical Papilla (SCAPs)

SCAPs are stem cells found at the apical papilla of the tooth and can be easily isolated from adult immature teeth with high proliferative and migratory potential. They secrete a broad variety of neurotrophic and regenerative growth factors and have immunomodulatory properties [44]. The study of Tatic et al. demonstrated the anti-inflammatory effect of ECM hydrogels in combination with SCAPs on microglial cell inflammation present after spinal cord injury. They proved that ECM hydrogels can deliver human mesenchymal stem cells from apical papilla and thus reduce local inflammation and provide a regenerative microenvironment [45].

One of the main intentions in cell therapy is promoting the survival of transplanted cells in the tissue over time. Factors like mechanical and nutritional stress and hypoxia as well as immune responses limit cell survival and characteristics after the transplantation process. Many attempts have been tested to increase cell homing including biomaterial or growth factor coadministration and preconditioning of stem cells [46]. Furthermore, it is necessary to focus on pharmacotherapy standardly administered to patients after stem cell implantation surgery, which is able to modulate the properties of the implanted stem cells.

## 6. Current NSAID Pharmacological Approaches for Dental Pain Treatment

Therapeutic management of sensitive and painful regions after surgery is an important aspect affecting the results of stem cell therapy. Current pharmacological management of pain arising from inflammation at the site of implantation utilizes several approaches; the most frequently used are anti-inflammatory drugs (NSAIDs), opioids, and N-methyl-d-aspartic acid (NMDA) receptor blockers [47].

First-line drugs used in the management of minor to moderate postoperative pain in dentistry are paracetamol and NSAIDs [48]. Both have been proven to be safe and effective, having them at the most favoured options among available treatments [49]. Paracetamol or acetaminophen is a very effective analgesic with very little anti-inflammation action. The exact mechanism in humans remains unclear, believed to modulate splice variant of cyclooxygenase 1 (COX-1) [50]. NSAIDs inhibit the production of prostaglandins (PGs) by blocking the activity of both cyclooxygenases 1 (COX-1) and 2 (COX-2). They act as nonselective inhibitors of the tissue COX, which catalyses the formation of prostaglandins and thromboxanes from arachidonic acid. Constitutively, the expression of COX-1 was proved in various tissues, whereas COX-2 is an inducible form occurring mostly in the kidneys and the central nervous system [51]. Results of COX-1 activity are the generation of prostaglandins necessary for human body function, like gastric mucosal integrity, platelet homeostasis, and regulation of renal blood flow. The expression of COX-2 is induced in inflammatory tissues by cytokines, lipopolysaccharides, and tumor necrosis factor *α*. The activity of COX-2 produces PGs with important roles in inflammation, cell proliferation, angiogenesis, invasiveness, extracellular matrix adhesion, immune response, and cell apoptosis. These proinflammatory prostaglandins mediate pain and inflammation processes, such as pulpitis, periodontitis, or pain following surgery. Thus, nonselective NSAIDs possess serious gastrointestinal adverse effects in long-term use, caused mainly by inhibition of COX-1. Selective COX-2 inhibitors, like rofecoxib and celecoxib, have improved safety profiles for gastric side effects. In addition, they depress prostacyclin, an atheroprotective agent, but not COX-1-related thromboxane, a proaggregatory agent and vasoconstrictor, which might predispose patients to heart attack and stroke [52].

**Figure 2:**
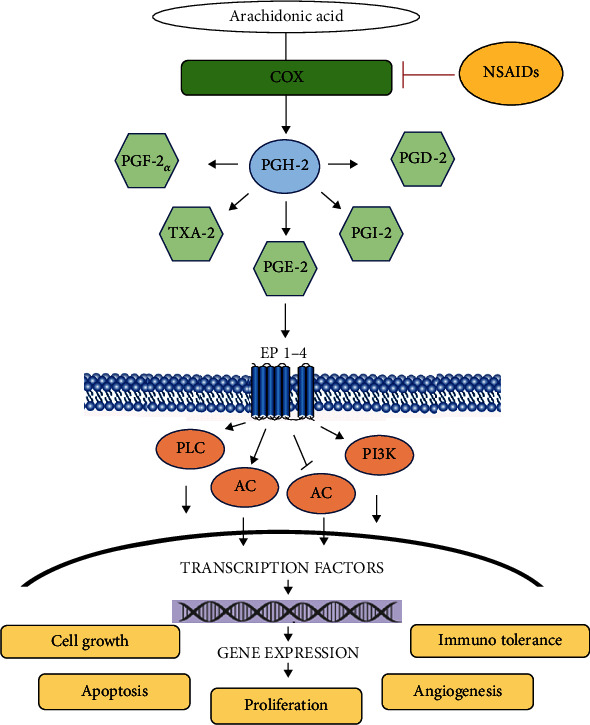
NSAID effect on COXs in prostaglandin E2 (PGE2) synthesis and pathways. Arachidonic acid is released from membrane phospholipids by phospholipase A2 and converted into PGH-2 by COXs. Activity of COXs, COX-1 and COX-2, is inhibited by NSAIDs. PGE2 is produced by PGE synthase and signals by binding to its G protein-coupled receptors EP1–EP4. Activation of EP1 (coupled to Gq) increases intracellular Ca2+ via phospholipase C (PLC). Activation of EP3 (coupled to Gi) increases intracellular Ca2+ via PLC and/or inhibits cAMP production via adenylate cyclase (AC). Activation of EP2 or EP4 (both coupled to Gs) stimulates cAMP production via AC. Activation of EP4 also increases protein kinase B (AKT/PKB) via stimulation of phosphoinositide 3-kinase (PI3K). Activation of G protein-coupled receptor triggers many signalling pathways and affects several transcription factors and gene expression levels.

Pain and inflammation are major concerns for patients undergoing surgery. Understanding the impact of anti-inflammatory drugs on transplanted stem cell functions is crucial for the healing process. Some studies revealed that NSAIDs have no significant cytotoxic effect on bone marrow stem cells from mice, while the proliferation suppressive effects occurred at concentration covering therapeutic doses (nonselective NSAIDs 10^−5^ M and COX-2 inhibitors 10^−6^ M). Their results suggest that the deteriorated effects of NSAIDs on mesenchymal stem cells are more likely due to the inhibition of cell proliferation than induction of cell death. Other studies indicated that NSAIDs suppress proliferation, arrest cell cycle, or induce apoptosis in vascular smooth muscle cells, colon cancer cells, and others [53]. The study of Almaawi et al. showed regulation of osteogenic and chondrogenic marker genes in MSC cells by NSAIDs and paracetamol, but no effect was observed in connection with diclofenac use [54]. This knowledge may help design better treatment strategies for stem cell implantation in different treatment approaches. Moreover, other ways of NSAID effect on engraftment and the fate of MSCs during transplantation have not yet been explored and should be investigated. Possible effects of NSAID on COXs in prostaglandin E2 are shown in Figure 2.

## 7. Summary

Most promising candidates for regenerative therapy of maxillofacial bone defects represent postnatal stem cells, as stem cells derived from dental tissue, because of their replication potential in an undifferentiated state and their ability to differentiate as well. One of the main intentions in cell therapy is promoting the survival of transplanted cells in the tissue over time. Factors like mechanical and nutritional stress and hypoxia as well as immune responses limit cell survival and characteristics after the transplantation process. Furthermore, it is necessary to focus on pharmacotherapy administered to patients after stem cell implantation surgery, which is able to modulate the properties of the implanted stem cells and thus influence the outcome of cell therapy used in the treatment of maxillofacial bone defects.
